# Comment on “Intoxication by hand sanitizer due to delirium after infectious spondylitis surgery during the COVID-19 pandemic: A case report and literature review”

**DOI:** 10.1016/j.ijscr.2020.11.122

**Published:** 2020-12-16

**Authors:** Nick Flynn

**Affiliations:** Department of Clinical Biochemistry, Cambridge University Hospitals NHS Foundation Trust, Hills Road, Cambridge, CB2 0QQ, United Kingdom

In a recent article in Int J Surg Case Rep [1], Lim refers to “creatinine kinase” (sic) in the article keywords.

Rather than “creatinine kinase”, presumably the author was instead referring to the enzyme creatine kinase. Creatine kinase (also known as creatine phosphokinase) catalyses the reversible phosphorylation of creatine to phosphocreatine, is frequently measured as a marker of muscle damage, and is commonly abbreviated as “CK” [2]. Creatinine is neither a product nor substrate for creatine kinase and is instead formed from creatine and phosphocreatine via non-enzymatic reactions.

The mistake of referring to creatine kinase as creatinine kinase is common in the medical literature. It is likely that most readers understood the author as they intended. However, this misspelling has the potential to cause confusion, can complicate literature searches, and may result in a loss of reader confidence in what might otherwise be a high-quality publication.

## Declaration of competing interest

None.

## Funding

None.

## Ethical approval

Not applicable.

## Consent

Not applicable.

## Author contribution

Nick Flynn is sole author.

## Registration of research studies

Not applicable.

## Guarantor

Nick Flynn.

## Provenance and peer review

Not commissioned, externally peer-reviewed.
